# Comparative Effects of Liquid and Solid Concentrated Growth Factors, and Injectable and Advanced Platelet-rich Fibrin on Proliferation and Differentiation of Human Dental Pulp Stem Cells: Comparative Effects on Proliferation and Differentiation of Human Dental Pulp Stem Cells

**DOI:** 10.31661/gmj.v13iSP1.3755

**Published:** 2024-12-29

**Authors:** Mohammadreza Talebi Ardakani, Sarah Noorizadeh, Mohammad Hossein Talebi, Amir Talebi, Alireza Azadi Hossein Abad

**Affiliations:** ^1^ Department of Periodontics, School of Dentistry, Shahid Beheshti University of Medical Sciences, Tehran, Iran; ^2^ Department of Periodontics, Faculty of Dentistry, Shahed University, Tehran, Iran; ^3^ Queen’s Belfast Dental School, Belfast, Northern Ireland; ^4^ Dental School, Universidad European de Madrid, Madrid, Spain

**Keywords:** Dental Pulp, Stem Cells, Platelet-Rich Fibrin

## Abstract

**Background:**

This study compared the effects of liquid (L-CGF) and solid (S-CGF) concentrated growth factors, and injectable (I-PRF) and advanced (A-PRF) platelet-rich fibrin on the proliferation and differentiation of human dental pulp stem cells (DPSCs).

**Materials and Methods:**

Blood samples were collected to prepare A-PRF, I-PRF, L-CGF, and S-CGF according to the standard protocols. DPSCs were exposed to the biomaterials, and their proliferation was quantified after 24 hours and 5 days using the methyl thiazolyl tetrazolium (MTT) assay. Cell differentiation was histologically assessed by Alizarin red staining. Expression of osteocalcin (OCN), osteopontin (OPN), and RUNX2 genes was assessed in non-osteogenic medium, and osteogenic medium after 7 and 14 days by real-time polymerase chain reaction (PCR).

**Results:**

The mean cell proliferation was not significantly different among the study groups (P=0.324) and did not significantly change over time (P=0.346). S-CGF, L-CGF, and A-PRF showed a significant difference in OCN expression (P0.001). The mean expression of OCN at 7 days was significantly lower in non-osteogenic medium. The mean expression of OCN and OPN at 7 days was significantly lower than 14 days. The mean expression of OPN at different time points was significantly different in A-PRF (P0.002), L-CGF (P0.003), and I-PRF (P0.003) groups. The mean expression of RUNX2 was significantly different at different times in L-CGF group, and a significant difference existed in the expression of RUNX2 in non-osteogenic medium and at 14 days.

**Conclusion:**

Within the limitations of the present study, the results showed that A-PRF, I-PRF, L-CGF, and S-CGF can increase the proliferation and differentiation of human DPSCs by regulating gene expression, and can be suitable options for osteogenesis. There was no significant difference in terms of mean cell proliferation among the study groups.

## Introduction

Periodontitis is a complex multifactorial disease that can lead to connective tissue attachment loss and periodontal tissue destruction. The main goal of periodontal therapy is to prevent disease progression and induce regeneration of the destructed tissues. Periodontal regeneration is a complex process that includes several biological phases such as cell adhesion, migration, proliferation, and differentiation in a synchronized sequence [1].

Tissue healing is guided by signaling proteins. Although understanding of this process at the microcellular level is still incomplete [2], evidence shows that growth factors and cytokines released from platelets play a fundamental role in the process of inflammation and wound healing [3]. Platelet-rich plasma (PRP) is a concentrated volume of autologous human platelets that contain human growth factors such as platelet-derived growth factor (PDGF) and transforming growth factors 1 and 2 (TGF1 and TGF2) [4][5].

The successful application of PRP alone or in combination with other graft materials has been previously documented [6]. However, the main drawback of PRP is presence of anticoagulants in its composition, which can interfere with the normal healing process [7].

Platelet-rich fibrin (PRF) is a second-generation platelet concentrate, which is prepared with no anticoagulant or activator [6]. PRF is superior to other platelet concentrates such as PRP due to its lower cost, easy preparation, and not requiring any additive [8].

Recently, a modified form of PRF known as advanced-PRF (A-PRF) with different mechanical properties was introduced, which appears to have higher platelet and growth factor content due to the lower speed of centrifugation. This 3D fibrin network has no cytotoxic potential that can structurally mimic the extracellular matrix and provide a suitable scaffold for optimal cell function [9]. Another new formulation of PRF known as injectable-PRF (I-PRF) was also introduced, which has a high volume of growth factors due to the lower speed and shorter duration of centrifugation [10]. Concentrated growth factors (CGF) [11] have a harder fibrin structure than PRP and PRF with a high content of growth factors.

CD34 is an intramembranous phospho-glycoprotein, which is primarily expressed by cells in the hematopoietic and vascular tissues. Although information regarding its precise function is limited [12], CD34+ cells are often used clinically to quantify the number of hematopoietic stem cells for grafting [13]. Stem cells are increasingly used as a good source of cells for tissue engineering due to their easy availability, high division rate, and optimal regeneration potential. They can be isolated from different oral tissues such as periodontal ligament or dental pulp, and have shown promising results in osteogenesis [14]. Thus, this study aimed to compare the effects of I-PRF, A-PRF, L-CGF, and S-CGF on proliferation and differentiation of human dental pulp stem cells (DPSCs) and expression of osteogenic genes.

## Materials and Methods

**Table:** Table1. Primer Sequences for GADPH, RUNX2, OPN, and OCN Genes

**Gene**	**Sequence of primers (5’ to the 3’)**	**Length**	**Product size**
**GAPDH**	Forward: CAT CAA GAA GGT GAA GCA G Reverse: GCG TCA AAG GTG GAG GAG TG	22 20	120
**RUNX2**	Forward: CGG AAT GCC TCT GCT GTT ATG Reverse: ACG ATT TGT GAA GAC GGT TAT GG	21 23	117
**OPN**	Forward: TGG TCA CTG ATT TTC CCA Reverse: TAT CAC CTC GGC CAT CAT	19 18	90
**OCN**	Forward: CTC ACA CTC CTC GCC CTA TTG Reverse: GTC AGC CAA CTC GTC ACA G	21 19	246

This in vitro, experimental study was conducted after gaining approval from the ethics committee of Shahid Beheshti University of Medical Sciences (IR.SBMU.DRC.REC.1399.100). The sample size was calculated to be 3 participants for each of the I-PRF, A-PRF, L-CGF, and S-CGF groups based on a previous study [?].

To prepare L-CGF, S-CGF, A-PRF, and I-PRF, blood samples were collected from candidates after obtaining their written informed consent. The candidates were systemically healthy non-smokers whose blood factors including platelet and leukocyte count were normal according to their peripheral blood test, and had no history of taking aspirin or any other medication that can affect blood coagulation in the past 2 weeks.

### Preparation of I-PRF

A total of 10 mL of whole blood was centrifuged at 700 rpm for 3 minutes without any anticoagulant.

### Preparation of A-PRF

A total of 10 mL of whole blood was centrifuged at 1300 rpm for 8 minutes without any anticoagulant.

### Preparation of CGF

A total of 5 mL of whole blood with no anticoagulant was immediately centrifuged (MF200, Medifuge) for 13 minutes.

In these 3 groups, due to the absence of anticoagulant, a fibrin clot was formed in the middle of the glass tube between the red blood cells at the bottom and acellular plasma at the top.

### L-CGF

After centrifugation with Medifuge CGF blood phase separator, a liquid phase was obtained which had to be used within 20 to 30 minutes; otherwise, it would undergo gelation.

### S-CGF

After centrifugation with Medifuge CGF blood phase separator, a platelet gel fibrin clot was obtained.

### Cell Culture

Human DPSCs (98-DP-1-5) were purchased from the cell bank of the Dental Material Research Center of Shahid Beheshti University of Medical Sciences. After defrosting, they were incubated in 75-cm2 flasks (T75) in Dulbecco’s modified Eagle’s medium (DMEM) enriched with 15% fetal bovine serum (FBS; Gibco), and a mixture of 5% penicillin-streptomycin (Gibco) at 37°C and 5% CO2. At 24 hours after the attachment of cells to the bottom of the flask, the culture medium was replaced with 1% FBS and after 24 hours of incubation, the cells were exposed to the biomaterials and cultured in presence of 5% CO2 and 95% humidity at 37°C under a sterile cell culture hood. After the cell proliferation reached 80% confluence in the flask, the cells were passaged by 0.25% trypsin and 0.02% EDTA several times. The cells in the logarithmic phase were used for preparation of cell suspensions. Cell suspension with a density of 10,000 cells per well in a 96-well plate was used for the methyl thiazolyl tetrazolium (MTT) assay. The plates were incubated at 37°C and 95% humidity for 24 hours.

### Quantitative Assessment of Cell Viability and Proliferation by the MTT Assay

The MTT assay was used to assess the effects of L-CGF, S-CGF, A-PRF, and I-PRF on cell viability and proliferation 24 hours and 5 days after treatment. At each time point, one sample of each group was removed from the incubator, the culture medium was extracted under a biologic hood, and replaced with 200 µL of 10% MTT yellow solution. The plate was then covered with aluminum foil and incubated for 2 to 4 hours. After ensuring the formation of formazan crystals under an inverted microscope, the medium of each well was removed, and 200 µL of dimethyl sulfoxide solvent was added to each well to dissolve the crystals which resulted in appearance of a purple color. Subsequently, 100 µL of each well was added to the 96-well plate of ELISA-Reader, and the spectrophotometric absorbance (optical density) of the reduced MTT was read at 570 and 620 nm wavelengths.

### Quantitative Assessment of Gene Expression by Real-time Polymerase Chain Reaction (PCR)

For quantitative assessment of the expression of osteogenic genes, the cells were plated in 12-well plates at a density of 50,000 cells/well. All experimental groups were exposed to an osteogenic medium, and gene expression was quantified in non-osteogenic medium (DMEM with 1% antibiotic) and after 7 and 14 days in an osteogenic medium (DMEM with 100 µL M-ascorbate, and 10-7M dexamethasone).

The groups in the non-osteogenic medium were as follows:

Control group: DPSCs in non-osteogenic medium containing 15% FBS

I-PRF group: DPSCs in non-osteogenic medium containing 15% I-PRF

A-PRF group: DPSCs in non-osteogenic medium containing 15% A-PRF

L-CGF group: DPSCs in non-osteogenic medium containing 15% L-CGF

S-CGF group: DPSCs in non-osteogenic medium containing 15% S-CGF

The groups in osteogenic medium evaluated at 7 and 14 days were as follows:

Control group: DPSCs in osteogenic medium containing 15% FBS

I-PRF group: DPSCs in osteogenic medium containing 15% I-PRF

A-PRF group: DPSCs in osteogenic medium containing 15% A-PRF

L-CGF group: DPSCs in osteogenic medium containing 15% L-CGF

S-CGF group: DPSCs in osteogenic medium containing 15% S-CGF

The primer for GAPDH house-keeping gene was designed. RNA extraction, cDNA synthesis, and amplification were performed; expression of osteocalcin (OCN), osteopontin (OPN), and RUNX2 genes was also quantified by the delta-CT method.

### RNA Extraction

The cell monolayer was rinsed with cold phosphate-buffered saline, and 1 mL of lysis buffer (TRIzol) was added per 10 cm3 of the cell culture plate. The homogenized samples were stored at room temperature for 5 minutes, centrifuged, and the supernatant was collected and transferred to a new tube. Per each 1 mL of TRIzol, 0.2 mL of chloroform was added, vortexed for 15 seconds, and stored at room temperature for 2-3 minutes. The samples were then centrifuged at 2-8°C for 15 minutes. Then, the supernatant was carefully separated from the middle phase and transferred into a new tube. Per each 1 mL of TRIzol used in step 1, 0.5 mL of isopropyl alcohol was added and stored at 30°C for 10 minutes. The mixture was then centrifuged at 2-4°C for 10 minutes. After centrifugation, the RNA was precipitated at the bottom of the tube in the form of a gel. The supernatant was removed, and the RNA sediment was rinsed by adding 1 mL of 75% ethanol per each 1 mL of TRIzol during homogenization. This mixture was centrifuged at 7500 g for 5 minutes at 2-8°C. The remaining ethanol was removed. The RNA sediment was completely dissolved in DEPC-treated water in 1/40 ratio. The RNA concentration was quantified by a spectrophotometer (Thermo Scientific NanoDrop 2000, Canada) at 260 and 280 nm wavelengths.

### Synthesis of cDNA

Similar concentrations of RNA were added to 200-µL microtubes, and 1 µL of oligo (dT) and 1 µm of random hexamer primer were added. The microtubes were then filled with DEPC to reach the final volume of 4.13 µL. They were briefly centrifuged, stored at 70°C for 5 minutes, and placed on ice, and 4 µL of 5X first-strand buffer, 1 µL of dNTPs, 0.5 µL of RNasein, and 1 µL of M-MLV were added to them and centrifuged.

The microtubes were placed in a thermocycler for cDNA synthesis, and reverse transcription reaction was performed in a thermocycler (Peqlab, USA). The microtubes were then stored at -20°C for later use in real-time PCR.

### Real-time PCR

Table-1 presents the primer sequences for GADPH, RUNX2, OPN, and OCN genes. Pre-designed primers were obtained from the Dental Science Research Center. Master Mix Ampliqon was used for PCR (Light Cycler 96, Roche, USA). The required amounts of forward (1 µL) and backward (1 µL) primers and other components such as Master Mix (5 µL), distilled water (3 µL), and cDNA (1 µL) for each gene were added, and the final volume for all genes was reached to 10 µL.

### Statistical Analysis

The MTT data were compared among the groups using two-way ANOVA. Gene expression was compared in each group over time using repeated measures ANOVA and among the groups using ANOVA. The Bonferroni and Tukey’s post-hoc tests were applied for pairwise comparisons. All statistical analyses were performed using SPSS version 20 at a 0.05 level of significance.

## Results

**Table:** Table2. Mean Expression of OCN Gene in Non-osteogenic Medium and also in Osteogenic Medium at 7 and 14 Days

Group	Time	Mean	Std. deviation	P-value
	OCN non	.648	.056	
A-PRF	OCN 7d	.115	.022	.001
	OCN 14d	.518	.084	
	OCN non	.482	.262	
I-PRF	OCN 7d	.151	.045	.098
	OCN 14d	.444	.052	
	OCN non	1.217	.073	
L-CGF	OCN 7d	.195	.057	<.001
	OCN 14d	.730	.046	
	OCN non	2.180	.277	
S-CGF	OCN 7d	.185	.046	<.001
	OCN 14d	.830	.143	

non: Non-osteogenic medium

**Table:** Table3. Pairwise Comparisons of the Expression of OCN Gene in each Group at Different Time Points

Growth factor	(I) Time	(J) Time	Mean difference	P-value
	OCN non	OCN 7d	.532 ^*^	.021
A-PRF		OCN 14d	.129	.469
	OCN 7d	OCN non	-.532 ^*^	.021
		OCN 14d	-.403 ^*^	.035
	OCN non	OCN 7d	1.022 ^*^	.008
L-CGF		OCN 14d	.487 ^*^	.026
	OCN 7d	OCN non	-1.022 ^*^	.008
		OCN 14d	-.535 ^*^	.001
	OCN non	OCN 7d	1.995 ^*^	.014
S-CGF		OCN 14d	1.350 ^*^	.049
	OCN 7d	OCN non	-1.995 ^*^	.014
		OCN 14d	-.644 ^*^	.043

non: Non-osteogenic medium; ^*^ significantly different

**Table:** Table4. Mean Expression of OPN Gene in Non-osteogenic Medium and also in Osteogenic Medium at 7 and 14 Days

Group	Time	Mean	Std. deviation	P-value
	OPN non	.69	.06	
A-PRF	OPN 7d	.35	.06	.002
	OPN 14d	.26	.07	
	OPN non	.79	.23	
I-PRF	OPN 7d	.02	.00	.003
	OPN 14d	.63	.11	
	OPN non	.69	.05	
L-CGF	OPN 7d	.11	.02	.003
	OPN 14d	.66	.18	
	OPN non	.32	.11	
S-CGF	OPN 7d	.54	.15	.051
	OPN 14d	.73	.13	

non: Non-osteogenic medium

**Figure-1:**
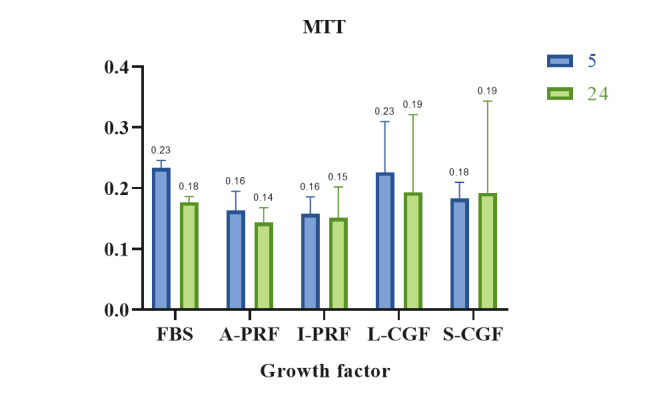
Figure 1.Optical density (proliferation) of the groups at 24 hours and 5 days

**Figure-2:**
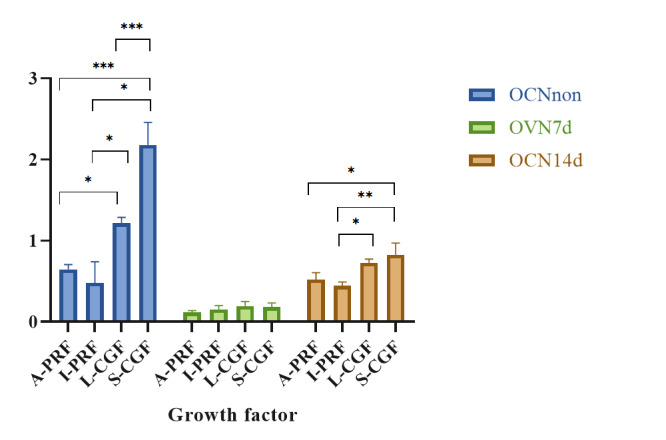
Figure 2. Pairwise comparisons of the four groups regarding the mean expression of OCN gene at different time points *Indicates presence of a significant difference.

**Figure-3:**
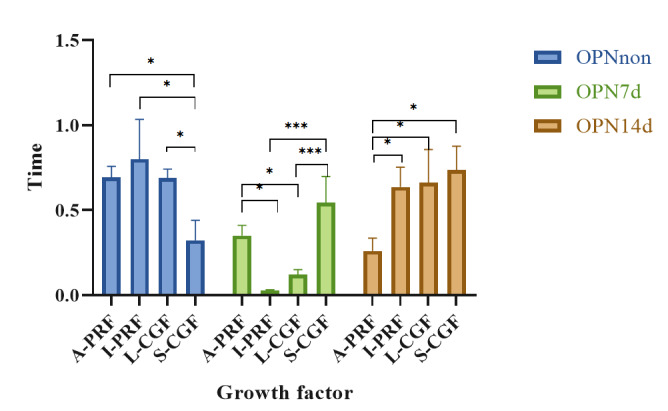
Figure 3. Pairwise comparisons of the groups regarding the mean expression of OPN gene at different time points *Indicates presence of a significant difference

**Figure-4:**
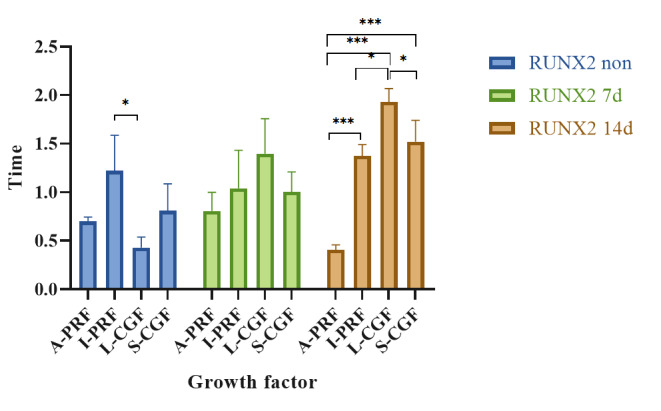
Figure 4. Pairwise comparisons of the groups regarding the mean expression of RUNX2 gene at different time points *Indicates presence of a significant difference.

### Results of Cell Proliferation Assessment by the MTT Assay

Figure-1 shows the optical density (cell proliferation) of the groups at 24 hours and 5 days. The results showed that the difference in the mean optical density was not significant among the groups (P=0.324). Also, the mean optical density at 24 hours and 5 days was not significantly different (P=0.346). The interaction effect of type of biomaterial and assessment time point on cell viability was not significant either (P=0.923).

### Results of the Assessment of OCN Gene Expression by Real-time PCR

Table-2 presents the mean expression of OCN gene in non-osteogenic medium and also in the osteogenic medium at 7 and 14 days. There was a significant difference in expression of OCN gene over time in S-CGF (P<0.001), L-CGF (P<0.001), and A-PRF (P=0.001) groups, but not in I-PRF group (P=0.098). The results of pairwise comparisons in each group are presented in Table-3.

There was a significant difference in the mean expression of OCN between the non-osteogenic and osteogenic media at 7 days in the APRF (P=0.021), L-CGF (P=0.008), and S-CGF (P=0.014) groups. However, only the L-CGF (P=0.026) and S-CGF (P=0.049) groups showed a significant difference between the non-osteogenic medium and osteogenic medium at 14 days. The difference in the mean expression of OCN in the osteogenic medium between 7 and 14 days was also significant in the APRF (P=0.035), L-CGF (P=0.001), and S-CGF (P=0.043) groups.

There was a significant difference in the mean expression of OCN gene among the three groups in non-osteogenic medium (P<0.00) and also at 14 days (P=0.003) but not at 7 days (P=0.200) in osteogenic medium (Figure-2). In non-osteogenic medium, the expression of OCN gene in the A-PRF group was significantly lower than that in the L-CGF (P=0.031) and S-CGF (P=0.000) groups. The mean OCN expression in the I-PRF group was significantly lower than that in the L-CGF (P=0.008) and S-CGF (P=0.000) groups. The mean OCN expression in the L-CGF group was significantly lower than that in the S-CGF group (P=0.001). Comparative results at 14 days in the osteogenic medium were as follows: the OCN gene expression in the A-PRF group was significantly lower than that in the S-CGF group (P=0.012), and the mean expression of OCN gene in I-PRF group was significantly lower than that in the L-CGF (P=0.020) and S-CGF (P=0.004) groups.

### Results of Assessment of OPN Gene Expression by Real-time PCR

Table-4 shows the mean expression of OPN gene in non-osteogenic medium and also in osteogenic medium at 7 and 14 days. The mean expression of OPN gene was significantly different at various time points in the L-CGF (P=0.003), I-PRF (P=0.003), and A-PRF (P=0.002) groups. The results of comparative evaluation showed that in the A-PRF group, the mean expression of OPN in non-osteogenic medium was significantly higher than that in osteogenic medium at 14 days (P=0.007). In the I-PRF group, the mean expression of OPN at 7 days was significantly lower than that at 14 days (P=0.034). In the L-CGF group, the mean expression of OPN in non-osteogenic medium was significantly higher than that in osteogenic medium at 7 days (P=0.011).

There was a significant difference in the expression of OPN gene among the four groups in non-osteogenic medium (P=0.013) and osteogenic medium at 7 (P<0.001) and 14 (P=0.011) days. Pairwise comparisons are presented in Figure-3. In the non-osteogenic medium, the mean expression of OPN in the A-PRF (P=0.043), I-PRF (P=0.012), and L-CGF (P=0.046) groups was significantly higher than the S-CGF group. In the osteogenic medium at 7 days, the mean expression of OPN in the A-PRF group was significantly higher than the I-PRF (P=0.007) and L-CGF (P=0.039) groups, and its mean expression in I-PRF was significantly lower than that in S-CGF group (P=0.000). At 14 days, the mean expression of OPN in the A-PRF group was significantly lower than the I-PRF (P=0.037), L-CGF (P=0.027), and S-CGF (P=0.011) groups.

### Results of Assessment of RUNX2 Gene Expression by Real-time PCR

Table-5 indicates the mean expression of RUNX2 gene in non-osteogenic medium and also in the osteogenic medium at 7 and 14 days. A significant difference was found in the mean expression of RUNX2 at different time points only in the L-CGF group (P=0.006). Pairwise comparisons in the L-CGF group revealed that the mean expression of RUNX2 in non-osteogenic medium was significantly lower than that in osteogenic medium at 14 days (P=0.012).

A comparison of RUNX2 expression among the four groups revealed significant differences in the non-osteogenic medium (P=0.022) and also in osteogenic medium at 14 days (P<0.001, Figure-4). It was indicated that in non-osteogenic medium, the mean expression of RUNX2 in the I-PRF group was significantly higher than that in the L-CGF group (P=0.016). At 14 days in osteogenic medium, the mean expression of RUNX2 in the A-PRF group was significantly lower than the I-PRF (P=0.000), L-CGF (P=0.000), and S-CGF (P=0.000) groups.

## Discussion

**Table:** Table5. Mean Expression of RUNX2 Gene in Non-osteogenic Medium and also in Osteogenic Medium at 7 and 14 Days

Group	Time	Mean	Std. deviation	P-value
	RUNX2 non	.70	.04	
A-PRF	RUNX2 7d	.80	.19	.105
	RUNX2 14d	.40	.05	
	RUNX2 non	1.21	.36	
I-PRF	RUNX2 7d	1.04	.39	.523
	RUNX2 14d	1.37	.11	
	RUNX2 non	.43	.11	
L-CGF	RUNX2 7d	1.39	.36	.006
	RUNX2 14d	1.92	.13	
	RUNX2 non	.81	.27	
S-CGF	RUNX2 7d	1.00	.20	.063
	RUNX2 14d	1.51	.21	

non: Non-osteogenic medium

This study compared the effects of I-PRF, A-PRF, L-CGF, and S-CGF on proliferation and differentiation of human DPSCs and expression of osteogenic genes.

The available literature on proliferation and differentiation of DPSCs only used the MTT assay, and evaluated the effects of A-PRF, I-PRF, or CGF on cell proliferation with no comparisons. However, in the present study, real-time PCR was also conducted in addition to MTT assay, and the biomaterials were compared regarding their effect on cell proliferation and osteogenic gene expression. Moreover, CGF was evaluated in solid and liquid forms. Since no previous study is available on the effect of the abovementioned biomaterials on human DPSCs using real-time PCR, exact comparison of results with previous findings is not possible.

The present results showed that the mean cell proliferation was not significantly different among the study groups, and did not significantly change over time. The highest rate of OCN gene expression in non-osteogenic medium and osteogenic medium at 14 days was recorded in the S-CGF group, while the L-CGF group indicated the maximum amount of expression in the osteogenic group at 7 days. The highest amount of OPN gene expression in non-osteogenic medium was recorded in the I-PRF group, while the S-CGF group indicated the maximum amount of expression in osteogenic group. The highest amount of RUNX2 gene expression in non-osteogenic medium was recorded in the I-PRF group, while the L-CGF group indicated the maximum amount of expression in the osteogenic group. However, the S-CGF, L-CGF, and A-PRF groups showed a significant difference in OCN, OPN, and RUNX2 gene expression.

Jin et al. [15] showed that the CGF scaffold had a fibrin network enriched with platelets and leukocytes, and had high biocompatibility with DPSCs. Higher cell proliferation was noted in the CGF groups in a dose-dependent manner. Compared with the control group, CGF groups with <50% concentrations increased cell migration, alkaline phosphatase activity, and deposition of mineralized tissue, while the cells exposed to higher doses (50% or 80%) indicated no significant difference. After induction of cell differentiation, the expression of dentin matrix protein-1, dentin sialophosphoprotein, vascular endothelial growth factor (VEGF) receptor-2, and cluster of differentiation (CD) 31 significantly increased in CGF groups compared with the control group. Moreover, endothelial cells derived from DPSCs induced by 5% CGF and VEGF resulted in the formation of maximum amount of mature tubular structures on Matrigel, although high-dose CGF showed no or inhibitory effect on cell differentiation. It has been confirmed that CGF has a complex internal structure, which can affect the release of growth factors and metabolites. Stem cells can regulate the production and release of CGF, show stemness properties, and differentiate into osteoblasts that produce mineralized matrix [16]. Moreover, it has been reported that CGF alone can induce osteogenic differentiation in human bone marrow stem cells. It also significantly increased the expression of proteins related to cell proliferation and migration. Thus, it can be used as a therapeutic protocol [17].

Growth factors regulate different behavioral, cellular, and molecular mechanisms and cause tissue regeneration as such. For instance, TGF-beta and insulin-like growth factor (IGF) cause cell proliferation. TGF-beta and VEGF increase cell migration. Bone morphogenetic proteins and fibroblast growth factor-2 induce osteogenic differentiation. VEGF and PDGF are also imperative for the process of angiogenesis, and VEGF increases the mitosis of vascular endothelial cells and controls the differentiation of mesenchymal cells by regulating RUNX2. [15]. Intrinsic growth factors are incorporated in the extracellular matrix, which are activated by the stimulation of cells and enhance cell proliferation [18]. I-PRF is injected into injured soft tissues, mucous membranes, or skin. For this purpose, blood is collected in glass tubes, centrifuged, and divided into two parts of (I) plasma-rich fibrin which contains leukocytes, platelets, and growth factors, and (II) red blood cells. Plasma is injected into the injured tissue and is penetrated deeper cutaneous layers using a derma pen, inducing the subsequent release of VEGF, epidermal growth factor, brain-derived growth factor, PDGF, TGF, and IGF [19]. A similar study [20] showed that I-PRF has a higher potential than PRP for the induction of human DPSCs. Moreover, I-PRF decreases the inflammatory conditions created by lipopolysaccharides and can induce regeneration of injured tissue by inducing odontoblastic differentiation of human DPSCs and reparative dentin formation. Also, I-PRF in 5% and 10% concentrations can significantly increase the proliferation and migration of mesenchymal stem cells [21]. The main mechanism of action of PRF is creation of a fibrin matrix for entrapment of platelets and subsequent release of cytokines [15]. Graziani et al. [22] showed that the optimal concentration of PRP which is effective for proliferation of osteoblasts is 2.5 times its concentration in blood serum, and cell proliferation decreases in presence of higher concentrations. Thus, in the present study, 1 mL of each of the A-PRF, I-PRF, L-CGF, and S-CGF biomaterials was added to 5 mL of DMEM because the amount of isolated platelet concentrate after centrifugation varies in different individuals based on age, gender, hematocrit, and blood factors. It can also be variable in several samples from the same patient. Also, the use of standard concentration of platelet products compared with their direct application has a technical advantage in the experimental phase and minimizes the required amount of blood.

A noteworthy finding of the present study was that over time (3 to 5 days), no significant increase occurred in cell proliferation, which is probably due to the early short-term significant effect of these extracts (within one day); thus, that the maximum proliferation capacity of the cells was reached in the first day probably due to the effect of VEGF, IGF, PDGF, and TGF-B, along with high proliferation potential of human DPSCs.

In the present study, in addition to assessment of cell proliferation, the expression of OCN, OPN, and RUNX2 osteogenic genes was also evaluated by real-time PCR. RUNX2 marker is the main gene responsible for osteogenic differentiation, and OCN and OPN are secondary and delayed markers of osteogenesis. Thus, expression of these three genes can help confirming the differentiation of human DPSCs to active osteoblasts. In the present study, the cells were exposed to 15% concentration of platelet extracts and also non-osteogenic medium for 5 days to assess their mutual proliferative-differentiation behavior.

Also, to confirm the osteogenic differentiation potential of stem cells, the control group cells were exposed to osteogenic medium for 7 and 14 days. The results showed that in non-osteogenic medium, S-CGF caused greater expression of OCN than other platelet products. Also, at 14 days in osteogenic medium, although OCN expression was lower than non-osteogenic medium, S-CGF still caused greater expression of OCN. Moreover, in the non-osteogenic medium, I-PRF caused greater expression of OPN while at 7 and 14 days in osteogenic medium, S-CGF caused greater expression of OPN. L-CGF caused a significantly lower expression of RUNX2 in non-osteogenic medium, while it caused maximum expression of RUNX2 at 14 days in osteogenic medium, which was statistically significant.

The study by Jin et al. [15] is the only available study similar to the present investigation concerning the type of stem cells. However, they only used the MTT assay while we also performed real-time PCR, which is a semi-quantitative method that enables comparison of the results with the findings of other studies.

However, the use of other molecular techniques to quantitatively compare gene expression among the groups can provide more detailed information regarding the differentiation behavior of stem cells.

The low level of I-PRF collected from some candidates, high contamination potential of culture media containing platelet concentrates over long periods, and high cost of real-time PCR for specific markers such as OCN were among the problems encountered in conduction of this study. In vitro design was a limitation of this investigation, which limits the generalization of the results to the clinical setting. Future well-designed studies are required over longer follow-ups. Also, simultaneous assessment of the pattern of release of growth factors from the fibrin scaffold of each platelet product is recommended.

## Conclusion

Within the limitations of the present study, the results showed that A-PRF, I-PRF, L-CGF, and S-CGF can increase the proliferation and differentiation of human DPSCs by regulating gene expression, and can be suitable options for osteogenesis. There was no significant difference in terms of mean cell proliferation among the study groups.

## Conflict of Interest

None.
